# Chemical analysis, inhibition of biofilm formation and biofilm eradication potential of *Euphorbia hirta* L. against clinical isolates and standard strains

**DOI:** 10.1186/1472-6882-13-346

**Published:** 2013-12-09

**Authors:** Shanmugapriya Perumal, Roziahanim Mahmud

**Affiliations:** 1 School of Pharmaceutical Sciences, Universiti Sains Malaysia, 11800 Minden, Penang, Malaysia

**Keywords:** Biofilm, Chemical composition, Ethnomedicine, Antibacterial, Anti-biofilm, Clinical isolate, Standard strain

## Abstract

**Background:**

The frequent occurrences of antibiotic-resistant biofilm forming pathogens have become global issue since various measures that had been taken to curb the situation led to failure. *Euphorbia hirta*, is a well-known ethnomedicinal plant of Malaysia with diverse biological activities. This plant has been used widely in traditional medicine for the treatment of gastrointestinal, bronchial and respiratory ailments caused by infectious agents.

**Methods:**

In the present study, chemical compositions of methanol extract of *E. hirta* L. aerial part was analyzed by gas chromatography and gas chromatography coupled to mass spectrometry. A relevant in vitro model was developed to assess the potency of the *E. hirta* extract to inhibit the bacterial biofilm formation as well as to eradicate the established biofilms. Besides biofilm, *E. hirta* extract was also evaluated for the inhibition efficacy on planktonic cells using tetrazolium microplate assay. For these purposes, a panel of clinically resistant pathogens and American type culture collection (ATCC) strains were used.

**Results:**

The methanolic extract of aerial part of *E. hirta* was predominantly composed of terpenoid (60.5%) which is often regarded as an active entity accountable for the membrane destruction and biofilm cell detachment. The highest antibacterial effect of crude *E. hirta* extract was observed in the clinical isolates of *Pseudomonas aeruginosa* with minimum inhibitory concentration (MIC) value of 0.062 mg/ml. The extract also displayed potent biofilm inhibition and eradication activity against *P. aeruginosa* with minimum biofilm inhibition concentration (MBIC) and minimum biofilm eradication concentration (MBEC) values of 0.25 mg/ml and 0.5 mg/ml, respectively.

**Conclusions:**

The crude methanol extract of *E. hirta* has proven to have interesting and potential anti-biofilm properties. The findings from this study will also help to establish a very promising anti-infective phytotherapeutical to be exploited in the pharmaceutical industries.

## Background

Ever since the advent of humanity on earth, plants have served unlimited source of phytotherapeuticals for various diseases [1]. In recent years, the emergence of biofilm infections have generated an urgent alarm in research and development field in seeks for novel antimicrobials from ethnomedicinal plant. National Institutes of Health (NIH) has estimated 70% of all microbial infections in the world are associated with biofilms [2]. Biofilms are developed by planktonic microorganisms aggregating together forming thin layer. Soon, close contact of few layers of microorganisms grow into dense, three dimensional structures accommodating millions of planktonic cells operating mutually to form a shield called biofilm. To date, the most common medically significant biofilm infections include eye, middle ear, urogenital tract, gastrointestinal tract, lung tissue and teeth [3]. Biofilms also accounted responsible for majority of contamination in the hospital devises and medical implants such as peritoneal membrane and dialysis catheters, indwelling catheters for chronic administration of chemotherapeutic agents, tracheal and ventilator tubing, prostheses, dental implants and cardiac implants [4]. Biofilms are far more difficult to treat as of bacteria living within are vastly resistant up to 1000 fold higher to potent antibacterial agents which are used as a last resort, including methicillin and vancomycin [5]. In contrast the same bacteria grown under planktonic forms are susceptible to those antibiotics.

*Euphorbia hirta* L., a member of the Euphorbiaceae family, is a common weed distributed in the temperate, sub-tropical, and tropical regions of the world. It is alternatively known by its Malay names as ‘ara tanah’ and ‘gelang susu’. This plant has been used in traditional Malay medicine for many years. The folk medicine used it in the treatment of gastrointestinal disorders, particularly intestinal parasitotosis, amoebic dysentery, diarrhoea, and ulcer [6,7]. The plant is also used in the treatment of bronchial and respiratory disorders including asthma, bronchitis, and hay fever. *E. hirta* is proven for well accepted pharmacological activities such as antioxidant [8], antibacterial [9,10], antifungal [11], diuretic [12], anthelmintic [13], antihypertensive [12], anxiolytic [14], antimalarial [15], anti-inflammatory [16], and anticancer [8].

Despite the various researches concerning the evaluation of multiple bioactivities, none have dealt with the bacterial biofilm investigation from *E. hirta*. Therefore, the aim of this study is to analyze the potency of *E. hirta* extracts as potential biofilm inhibitor and biofilm eradicator crude drugs as well as to comprehensively characterize the methanolic extract of *E. hirta* aerial part grown in Malaysia. A modified version of microdilution assay was developed in this investigation to measure the inhibition of biofilm formation and biofilm eradication activities from *E. hirta* L. against clinical isolates and standard strains.

From our literature search, there has been no comprehensive chemical composition data on the methanolic extract of *E. hirta* aerial part has been reported or evaluated. Moreover, the possibility is higher that of same species from different countries could show different chemical composition and biological activity. Therefore the present study is assumed the first to report fingerprinting profiles of *E. hirta* aerial part by GCMS-MS as well as the initial research to assess on biofilm inhibitory and eradication activity of this ethnomedicinal plant.

## Methods

### Chemicals and reagents

p-iodonitrotetrazolium violet (INT) were purchased from Sigma Chemical Co. (St. Louis, MO, USA). All other chemicals, namely dimethylsulfoxide (DMSO) and methanol were of analytical grade and obtained from Merck (Darmstadt, Germany).

### Plant collection and authentication

The fresh aerial parts of *Euphorbia hirta* L. were obtained from Relau, Penang City, Malaysia. The plant was collected during the period of July to August 2012. The plant was authenticated by Mr. Shunmugam, the botanist of the School of Biological Sciences, Universiti Sains Malaysia, where a voucher specimen (No.11254) was deposited in the Herbarium Unit of the school.

### Extraction of plant material

Aerial parts of *E. hirta* were air-dried and ground to mesh size No.40 and macerated solely with methanol by ratio of 10 g of ground plant material in 100 ml of methanol. Extraction was done for 6 days under occasional shaking and the process was repeated three times. The combined extracts obtained were filtered and concentrated to dryness with a rotary evaporator (Rotavapor® R-200, Buchi, Switzerland) under reduced pressure. The extract obtained was eventually freeze-dried (FreeZone®, MO) to remove any residual water. The yield of extract was calculated. The extractive procedure was performed in dim lighting and all the dried extracts were stored at 4°C until use. For GCMS analysis, sample was prepared by dissolving in methanol to obtain a concentration of 1 mg/ml. The sample was filtered through 0.22 μm syringe filter devices (Milipore) prior to injecting in the chromatography system.

### Gas chromatography (GC) and gas chromatography mass spectrometry (GC/MS) analysis

The methanol extract of *E. hirta* was analyzed by GC and GC–MS using an Agilent-Technologies 6890 N Network GC system equipped with an Agilent-Technologies 5973 MSD mass spectrometer and Agilent-Technologies 7683B series auto injector. The GC/MS was operated under the following conditions. Separation was performed on HP-5MS capillary column (30 m × 0.25 mm × film thickness 0.25 μm). The column temperature was programmed from 70°C to 280°C at a rate of 20°C /min with the lower and upper temperatures being held for 3 and 10 min, respectively. The GC injector and MS transfer line temperatures were set at 250°C and 280°C, respectively. GC was performed in the splitless mode. Helium was used as carrier gas at a flow rate of 1.1 ml/min. For MS detection, the electron ionization mode with ionization energy of 70 eV was used, with a mass range at m/z 30–650. An injection volume of 1 μl was used for the methanol extract. The components were identified by their retention times and based on the commercially available spectral, (National Institute of Standards and Technology (NIST) mass spectral search program for NIST/EPA/NIH Mass Spectral Library V2.0) and mass fragmentation patterns using data of standards at Wiley 7.0 Library. GC and GC–MS analyses were performed in triplicate.

### Bacterial strains

The susceptibility test comprises a panel of clinically resistant Gram-negative and Gram-positive bacteria. These selected human pathogenic bacteria are capable of forming biofilms and causes severe infections. The panel of clinically resistant pathogens used in this study includes *Klebsiella pneumonia, Pseudomonas aeruginosa, Salmonella typhi, Shigella dysenteriae, Enterobacter aerogens, Escherichia coli, Enterococcus faecalis, Proteus mirabilis, Proteus vulgaris*, *Bacillus subtilis* and *Bacillus cereus.* The clinical isolates were obtained from Department of Medical Microbiology and Parasitology (JTMP), School of Medical Sciences, Universiti Sains Malaysia, and stored at -80°C in tryptic soy broth containing 50% glycerol. Their identities were confirmed by biochemical test using API (analytical profile index) system. The bioassays was also performed in parallel with well characterized strains obtained from the American Type Culture Collection (ATCC), including *Escherichia coli* (ATCC 25922), *Staphylococcus aureus* (ATCC 25923), *Pseudomonas aeruginosa* (ATCC 27853), *Bacillus subtilis* (ATCC 6633), *Enterococcus faecalis* (ATCC 29212), methicillin-resistant *Staphylococcus aureus* (MRSA) (ATCC 33591), vancomycin-resistant *Enterococcus faecalis* (VRE) (ATCC 51299) and vancomycin-resistant *Enterococcus faecium* (VRE) (ATCC 700221). The ATCC strains used were able to produce biofilms in a given conditions. A stable non-biofilm producing clinical isolate *Staphylococcus aureus* (mutant) was used in the bioassays as control.

### Inocula preparation

All the bacterial strains were recovered on a fresh tryptic soy agar (Difco, USA) plate 24 h prior to antimicrobial test. To prepare the inoculum, colonies from fresh tryptic soy agar were transferred into sterile Mueller Hinton (MH) liquid growth medium and incubated at 37°C overnight. Aliquots (500 μl) were transferred to 10 ml of fresh MH broth and incubated at 37°C. The optical density 600 nm wavelength (OD_600_) was monitored until the exponential growth phase was reached. Cells were harvested by centrifugation (3,000 g, 5 min at 4°C), washed in 10 mmol/L phosphate-buffered saline (PBS) (pH 7.4), and resuspended in MH broth to an approximate cell density of 1.0 × 10^5^ CFU/ml. The final cells concentration was confirmed by viable counts.

### Minimum inhibitory concentration (MIC) and minimum bactericidal concentration (MBC)

The minimum inhibitory concentrations (MIC) of *E. hirta* were determined by using tetrazolium microplate assay which were slightly modified from serial broth microdilution method as described by Eloff [17]. This assay was performed using Corning 35–1172 flat-bottomed polystyrene 96-well clear microtitre plates with standard plate layout as proposed by Cos *et al. *[18]. Briefly, methanol extract of *E. hirta* were dissolved in DMSO and an identical two-fold serial dilution using MH broth were made to form 0.062 – 2.0 mg/ml. One hundred microlitres of bacterial inoculum was added and mixed thoroughly in all the wells (0.031 – 1.0 mg/ml as the highest in-test concentration. The microtitre plates were sealed with parafilm tape and incubated overnight at 37°C. An appropriate mixture of solvent DMSO, medium and inoculum were included as drug-free control and the final concentration of DMSO in the well was ensured to be less than 1% (v/v). Clinically established antibiotic, cefepime was used in parallel experiment as a positive drug control. An additional non-infected medium was included for sterility check. The MIC of *E. hirta* extract was detected following addition of 50 μl of INT (2-4-Iodophenyl-3-4-nitrophenyl-5-phenyl-2H-tetrazolium chloride) at a final concentration 0.2 mg/ml in all the wells and incubated for further 30 min at 37°C. Bacterial growth was determined by observing the color change of INT in the microplate wells. Biologically active bacterial cells will reduce the colourless tetrazolium salt which act as an electron acceptor to a red-coloured formazan product [19]. Inhibition of bacterial growth is observed when the solution in the well remained clear after incubation with INT. MIC was defined as the lowest extract concentration that completely inhibits the growth of microorganisms.

For the determination of minimum bactericidal concentration (MBC), 20 μl of culture medium from the microtitre plate wells that showed no changes in color will be re-inoculated on MH agar plates. After 24 h of incubation at 37°C, MBCs were determined as the lowest concentration that yielded nil bacterial growth on MH agar plates. The MIC and MBC determination were performed three times in duplicate.

### Inhibition of biofilm formation

The effect of *E. hirta* extract to inhibit biofilm formation was measured using microplate based assay, modified from Stepanovic *et al*. [20]. Briefly, the bacterial cells were grown in tryptic soy broth (TSB) at 37°C overnight. The cultures was then harvested by centrifugation (14,000 rpm, 15 min at 4°C) and rinsed with phosphate-buffered saline (PBS, pH 7.4). The washed bacterial cells were then resuspended in tryptic soy broth to approximately 1 × 10^7^ cfu/ml (determined by OD and plate count assay). Two-fold serial dilution of *E. hirta* extract was made with TSB to achieve concentrations ranging from 0.062 – 2.0 mg/ml. An amount of 40 μL of the *E. hirta* extract solution was then pre-mixed with the bacterial inocula (360 μL) to attain final concentration ranging from 0.031 – 1.0 mg/ml. One hundred microliters of this mixture for each concentration was added to three separate wells in the 96-well microplates for replicate testing. Wells containing mixture of dilute DMSO and inoculum were included as control with DMSO final concentration of 1% (v/v). After 24 h of incubation at 37°C, the supernatant containing TSB and planktonic cells were gently removed from the microplate wells and the wells were washed twice with 150 μL of PBS. After rinsing, 30 μl of the INT reagent were added and the suspension was incubated for 4 h at 37°C. The minimum biofilm inhibitory concentration (MBIC), defined as the lowest concentration of an antimicrobial agent required to inhibit the formation of biofilms was determined by observing the color change of INT in the microplate wells as described above. Non-biofilm forming clinical isolate *S. aureus* was used as negative control and wells containing clinically established drug (cefepime) were used as positive control. The bioassay was performed in triplicate.

### Eradication of biofilms

In order to test the ability of *E. hirta* extract to eradicate the formed biofilms, a modified microdilution assay described by LaPlante [21] was employed. Microbial biofilms were developed in flat bottom 96-well clear microtitre plates. A volume of 30 μl of a washed suspension of bacterial cells grown into the mid exponential phase in tryptic soy broth (1.0 × 10^5^ CFU/ml) were inoculated into fresh tryptic soy broth at 150 μL/well. The cultured microtitre plates were incubated at 37°C to permit the microbial cells to form the biofilms. After 24 hours of biofilm growth, the wells were rinsed with PBS (pH 7.4) to remove non-adherent cells. The biofilms established for 24 hours in the each well were subsequently treated with two-fold serial dilutions of *E. hirta* extract (1 mg/ml as the highest in-test concentration). Non-biofilm isolate was used as negative control and wells containing clinically established drugs were used as positive control. The microtitre plates were sealed with parafilm tape and incubated overnight at 37°C. The minimal biofilm eradication concentration (MBEC), defined as the lowest concentration of an antimicrobial agent required to eradicate biofilm, was determined by observing the color change of INT in the microplate wells as per MIC determination method. The bioassay was performed in triplicate.

### Statistical analyses

The results were analyzed using Statistical Package for the Social Sciences (SPSS) software version 17.0. All the values are expressed as means ± standard deviation (SD) from triplicate experiments.

## Results

### GC and GC–MS analyses

Analyses (GC and GC-MS) of aerial part of *E. hirta* methanol extract enabled the identification of nineteen chemical compounds showing more than 90% similarity with the standard mass spectra in the library. The qualitative analytical results are shown in Table 1. This extract is characterized by the presence of diterpenes 8.7%, triterpenes 16.0%, triterpenoids 12.9%, sterols 36.6%, tocols 1%, esters 9.2%, fatty acids 15.3%, and ketones 0.3%. The dominant compounds found in the extract were cycloartenol (34.7%), alpha-amyrin (10.7%), clionasterol (10.4%), phytol (8.5%), linoleic acid (7.7%), palmitic acid (7.2%) and 2-monopalmitin (4%). Other components were present in amounts less than 4%. Squalene and megastigmatrienone are being reported for the first from this spesies.

**Table 1:** **Chemical composition of methanol extract of *Euphorbia hirta***

**N0.**	**Compound**^**a**^	**RT**^**b**^	**Composition (%)**
1	Megastigmatrienone	6.711	0.1
2	6,10,14-Trimethyl-2-Pentadecanone	7.803	0.1
3	Methyl palmitate	8.202	1.1
4	Methyl ester of 3-(3,5-di-tert-butyl-4-hydroxyphenyl)-propionic acid	8.349	0.4
5	Palmitic acid	8.426	7.2
6	Methyl linolenate	9.071	1.9
7	Phytol	9.127	8.5
8	Linoleic acid	9.288	7.7
9	2-monopalmitin	10.856	4
10	2-monostearin	11.906	2.2
11	Squalene	12.628	2.2
12	Vitamin-E	15.645	1
13	Neophytadiene	15.849	0.2
14	Campesterol	17.06	1.9
15	Clionasterol	18.607	10.4
16	Alnulin or skimmiol	18.943	2.5
17	Beta-amyrin	19.273	3.1
18	Cycloartenol	20.078	34.7
19	Alpha-amyrin	20.295	10.7
	% of identification		99.9
	Terpene		0.1
	Diterpenes		0.2
	Triterpene		2.2
	Terpenoid		1.0
	Diterpenoid		8.5
	Triterpenoid		51.0
	Fatty acids		22.2
	Ester		2.3
	Sterol		12.3
	Other		0.1

^a^Compounds are listed in order of elution from a HP-5 MS column.

^b^Retention time (as minutes).

### Growth inhibition of planktonic cells

Initially, methanol extract of *E. hirta* was assessed on growth inhibitory ability against planktonic cells of clinical pathogens and standard strains. The potency was quantitatively assessed by the MIC and MBC values (Table 2). Among all the pathogen, *P. aeruginosa* was the most susceptible bacteria to *E. hirta* extract, with MIC and MBC values of 0.062 mg/ml and 0.125 mg/ml, respectively. A moderate growth inhibition was observed against *E. faecalis, Bacillus cereus and P. aeruginosa* (ATCC 27853) with MIC and MBC values of 0.125 mg/ml and 0.25 mg/ml, respectively. This extract also displayed mild antibacterial activities against *S. typhi, B. subtilis and S. aureus* (ATCC 25923) with MIC and MBC values of 0.25 mg/ml and 0.5 mg/ml, respectively. Compared to the positive control, cefepime belonging to the β-lactam antibiotics which is inhibitor of cell wall synthesis was found to possess high inhibitory activity against most of bacteria under investigation.

**Table 2:** **Biological activity of methanol extract of *E. hirta *** **(mg/mL) against resistant clinical isolates and standard strains**

**Bacteria**	**Methanol extract of *Euphorbia hirta***				**Cefepime**			
	**MIC**	**MBC**	**MBIC**	**MBEC**	**MIC**	**MBC**	**MBIC**	**MBEC**
**Resistant clinical isolates**								
**Biofilm-producing**								
*Klebsiella pneumonia*	1	>1	>1	>1	0.031	0.062	1	>1
*Pseudomonas aeruginosa*	0.062	0.125	0.25	0.5	0.015	0.031	0.125	0.5
*Salmonella typhi*	0.25	0.5	1	>1	0.062	0.125	0.5	>1
*Shigella dysenteriae*	1	>1	>1	>1	0.031	0.062	>1	>1
*Enterobacter aerogens*	1	>1	>1	>1	0.062	0.25	1	>1
*Escherichia coli*	0.5	>1	1	>1	0.015	0.031	0.5	1
*Enterococcus faecalis*	0.125	0.25	0.5	1	NA	NA	NA	NA
*Proteus mirabilis*	0.5	>1	>1	>1	0.031	0.125	>1	>1
*Proteus vulgaris*	0.5	>1	1	>1	0.062	0.25	1	>1
*Bacillus subtilis*	0.25	0.5	1	>1	0.031	0.125	>1	>1
*Bacillus cereus*	0.125	0.25	0.5	>1	NA	NA	NA	NA
**Non-biofilm producing**								
*Staphylococcus aureus*	0.125	0.25	0.25	0.5	0.015	0.031	0.5	1
**Standard strain**								
*Escherichia coli* (ATCC 25922)	0.5	1	>1	>1	0.015	0.031	0.5	1
*Staphylococcus aureus* (ATCC 25923)	0.25	0.5	1	>1	0.015	0.062	0.25	1
*Pseudomonas aeruginosa* (ATCC 27853)	0.125	0.25	0.5	1	0.031	0.062	0.5	1
*Bacillus subtilis* (ATCC 6633)	0.5	1	1	>1	0.125	0.5	1	>1
*Enterococcus faecalis* (ATCC 29212)	0.5	1	>1	>1	NA	NA	NA	NA
Methicillin-resistant *staphylococcus aureus*	0.5	1	1	>1	0.062	0.125	0.5	>1
(MRSA) (ATCC 33591)								
Vancomycin-resistant *enterococcus faecalis*	0.5	>1	1	>1	NA	NA	NA	NA
(VRE) (ATCC 51299)								
Vancomycin-resistant *enterococcus faecium*	1	>1	>1	>1	0.125	0.25	>1	>1
(VRE) (ATCC 700221)								

^a^Values expressed are mean ± standard deviation of three experiments; values given as mg/ml.

^b^NA, not active.

### Biofilm inhibitory activities of *E. hirta* extract

The capability of methanol extract of *E. hirta* to attenuate biofilm formation was shown in Table 2. The potent biofilm inhibition activity was detected against clinical isolate of *P. aeruginosa* with MBIC value of 0.25 mg/ml. A good inhibition of adherence was observed in *E. faecalis* and *P. aeruginosa* (ATCC 27853) with MBIC value of 0.5 mg/ml. The remainder of the biofilms recorded the lowest resistance to this methanol extract (MBIC ≥ 1). Within the tested MBIC range (0.031 to 1 mg/ml), cefepime showed weak biofilm inhibitory activity against all the pathogens.

### Eradication of the established biofilm by *E. hirta* extract

*E. hirta* extract effectively eradicated the established biofilm of *P. aeruginosa*, with minimum biofilm eradication concentration (MBEC) value of 0.5 mg/ml. The methanol extract also showed weak anti-biofilm activities against *E. faecalis* and *P. aeruginosa* (ATCC 27853) exhibiting MBEC value of 1.0 mg/ml. Nevertheless, *E. hirta* extract displayed no capability to disrupt the established biofilm in the rest of the selected pathogens at the highest test concentration of 1 mg/ml. Cefepime found to be ineffective to eradicate biofilm of all the tested pathogens except for the clinical isolate, *P. aeruginosa* with MBEC value of 0.5 mg/ml.

## Discussion

Biofilm infections represent a serious health threats worldwide today mostly due to the appearance of antibiotic resistant strains. Contemporary testing on minimum inhibitory concentration (MIC) which measures only planktonic susceptibility may be the possible explanation for treatment failures and resistant development among bacterial biofilms. In the present study, the results of the MIC, MBC, MBIC and MBEC have highlighted the interesting activity of *E. hirta*. The phytochemicals present in the crude methanolic extract of *E. hirta* plays an important role for the evident antibacterial and anti-biofilm activity. Medicinal plants are rich of secondary metabolites which some of them are directly involved in plant defense mechanisms against microorganisms [22]. In the current investigation, GC-MS analysis of methanol extract of *E. hirta* aerial part was conducted with the intention to correlate the phytochemical compounds to the antibacterial and anti-biofilm activity. The GC-MS profile of methanol extract of *E. hirta* revealed the presence of nineteen compounds. The most abundant phytocompound found was terpenoids. Terpenoids are the largest group of natural plant products many of which well acknowledged as potential antimicrobial agents. It is feasible that the observed broad spectrum antibacterial activity by methanolic extract of *E. hirta* probably attributed to the dominant presence of some terpenoids (cycloartenol, squalene, α-amyrin and β-amyrin). Previous studies have shown α-amyrin and β-amyrin were potential to inhibit the growth of some microbes [23,24]. These terpenoids most likely involved in the detachment of planktonic cells from the biofilm. Probably the terpenoids influenced the membrane integrity in all organisms and helped to eradicate most biofilm cells. The significant reduction in cell attachment made terpenoids an ideal anti-adhesive compound. Terpenes have also been frequently reported to be active against bacteria [25]. The mechanism of action of terpenes is thought to involve membrane disruption by the lipophilic compounds and thereby inhibit respiration and ion transport processes in the bacterial cells [22]. De Carvalho *et al., *[26] proved that natural compounds such as terpenes can be used to prevent cell aggregation and biofilm formation. Terpenes are believed to influence the fatty acid composition of the cell membrane, and thus cell hydrophobicity which lead to the eradication of the biofilm. Additionally, the expression of synergy, antagonism or additive effects among the major phytocompounds found in the crude extract may also be the rationale to the apparent anti-biofilm activity. The standard antibiotic (cefepime) used in this study showed potent antibacterial and anti-biofilm activity due to the fact that they appeared in purest form in contrast to the crude extract which is in complex mixtures of compounds. The MIC of the standard antibiotic used as positive control is given in Table 2. GC-MS analysis helps towards comprehending the phytocompounds with remedial values in *E. hirta*.

To begin our investigation on antibacterial activities, the growth inhibition effect of *E. hirta* methanolic extract were determined first on planktonic cultures of resistant clinical isolates and standard strains. *E. hirta* extract exerted broad antibacterial spectrum with substantial potency against both Gram-positive and Gram-negative bacteria tested. The standard strains presented more invulnerability towards *E. hirta* methanol extracts compared to resistant clinical isolates. This is due to ATCC strains are well characterized cultures with higher stability towards the antimicrobials. The strongest inhibition was recorded against Gram negative, *P. aeruginosa* followed by moderate antibacterial activity against Gram positive, *E. faecalis* and *B. cereus.* The different cell wall susceptibility amongst bacteria may be the key contributor to various MIC and MBIC values. According to Fennel *et al., *[27], Gram positive bacteria are often found to be more susceptible to plant extracts than the Gram negative bacteria. It is well known that the outer membrane present only in the Gram negative bacteria play an important role as an effective barrier. However in this study, the prominent sensitivity of *P. aeruginosa* toward methanolic extract of *E. hirta* may possibly due to the membrane permeability. Similar results for the effect of essential oils on outer membrane permeability in Gram-negative bacteria were reported by Helander *et al., *[28]. Although Gram positive bacteria lack of outer membrane, the thicker cell wall consist of few peptidoglycan layers could act as functional barrier thus hinder the penetration of antimicrobial compound into the bacterial cell [29]. *E. hirta* extract displayed a distinct bactericidal mode of action against most of the bacteria tested. The bactericidal activity is confirmed by the obtained MBC values which are usually two to four higher than the corresponding MICs. The MIC and MBC values reported in this work were lower than those obtained from other studies involving *E. hirta* plant [10,30].

In contrast to the growth inhibitory ability against planktonic cells, biofilms of *P. aeruginosa* were less susceptible to *E. hirta* extract with MBIC value of 0.25 mg/ml. Bacteria living as biofilm are often more difficult to eradicate compared to the planktonic mode of growth. Planktonic cells forms biofilms by adhering to each other strongly via formation of pili. Apparently, in this study, the pilicides action of *E. hirta* methanolic extract might be the reason for the growth inhibition of *P. aeruginosa* biofilm. Besides pili formation, bacteria also use quorum sensing to coordinate the formation of biofilms. Quorum sensing (QS) is a cell-to-cell signaling mechanism which often linked to the establishment of complex communities of bacteria. The QS present between the bacterial inhabitants has led to development of the biofilms [31]. The opportunistic pathogen *P. aeruginosa* uses quorum sensing to coordinate the formation of biofilms, swarming motility, exopolysaccharide production, and cell aggregation [32]. The eradication of *P. aeruginosa* biofilm was interpreted to suggest that methanolic extract of *E. hirta* displays QS inhibitory activity. MBIC and MBEC values of *E. hirta* methanol extract against clinical isolates and standard strains are being documented for the first time in this study. This study outlines a very sturdy basis for future investigations in pursuit to discover new anti-infectious agents.

## Conclusions

In conclusion, the findings from this study seemed to validate the traditional use of *E. hirta* plant for the treatment of ailments caused by infectious agents. The interesting biofilm inhibitory and eradication activity found against *P. aeruginosa* biofilm makes this ethnomedicinal plant an outstanding candidate for nosocomial infection therapy. Moreover, *E. hirta* plant may also benefit the hospitals and healthcare facilities as biofilm control agents for the prevention of contamination in the medical devises. The antibacterial and anti-biofilm activities of crude extract of *E. hirta* plant could be further improved after the opportune bioactivity guided fractionation steps. This pace may help to discover and develop distinctive chemical entities with above promising biological activities.

## Abbreviations

GC: Gas chromatography; GC/MS: Gas chromatography mass spectrometry; MH: Mueller Hinton; PBS: Phosphate-buffered saline; MIC: Minimum inhibitory concentration; MBC: Minimum bactericidal concentration; MBIC: Minimum biofilm inhibitory concentration; MBEC: Minimal biofilm eradication concentration; INT: P-iodonitrotetrazolium violet; DMSO: Dimethylsulfoxide; ATCC: American type culture collection.

## Competing interests

The authors declare that no competing interest exists.

## Authors’ contributions

SP carried out the study, RM conceived of the study, supervised the work. Both authors read and approved the final manuscript.

## Pre-publication history

The pre-publication history for this paper can be accessed here:

http://www.biomedcentral.com/1472-6882/13/346/prepub
